# Neural Machine Translation–Based Automated Current Procedural Terminology Classification System Using Procedure Text: Development and Validation Study

**DOI:** 10.2196/22461

**Published:** 2021-05-26

**Authors:** Hyeon Joo, Michael Burns, Sai Saradha Kalidaikurichi Lakshmanan, Yaokun Hu, V G Vinod Vydiswaran

**Affiliations:** 1 Department of Learning Health Sciences University of Michigan Ann Arbor, MI United States; 2 Department of Anesthesiology University of Michigan Ann Arbor, MI United States; 3 School of Information University of Michigan Ann Arbor, MI United States

**Keywords:** CPT classification, natural language processing, machine learning, neural machine translation

## Abstract

**Background:**

Administrative costs for billing and insurance-related activities in the United States are substantial. One critical cause of the high overhead of administrative costs is medical billing errors. With advanced deep learning techniques, developing advanced models to predict hospital and professional billing codes has become feasible. These models can be used for administrative cost reduction and billing process improvements.

**Objective:**

In this study, we aim to develop an automated anesthesiology current procedural terminology (CPT) prediction system that translates manually entered surgical procedure text into standard forms using neural machine translation (NMT) techniques. The standard forms are calculated using similarity scores to predict the most appropriate CPT codes. Although this system aims to enhance medical billing coding accuracy to reduce administrative costs, we compare its performance with that of previously developed machine learning algorithms.

**Methods:**

We collected and analyzed all operative procedures performed at Michigan Medicine between January 2017 and June 2019 (2.5 years). The first 2 years of data were used to train and validate the existing models and compare the results from the NMT-based model. Data from 2019 (6-month follow-up period) were then used to measure the accuracy of the CPT code prediction. Three experimental settings were designed with different data types to evaluate the models. Experiment 1 used the surgical procedure text entered manually in the electronic health record. Experiment 2 used preprocessing of the procedure text. Experiment 3 used preprocessing of the combined procedure text and preoperative diagnoses. The NMT-based model was compared with the support vector machine (SVM) and long short-term memory (LSTM) models.

**Results:**

The NMT model yielded the highest top-1 accuracy in experiments 1 and 2 at 81.64% and 81.71% compared with the SVM model (81.19% and 81.27%, respectively) and the LSTM model (80.96% and 81.07%, respectively). The SVM model yielded the highest top-1 accuracy of 84.30% in experiment 3, followed by the LSTM model (83.70%) and the NMT model (82.80%). In experiment 3, the addition of preoperative diagnoses showed 3.7%, 3.2%, and 1.3% increases in the SVM, LSTM, and NMT models in top-1 accuracy over those in experiment 2, respectively. For top-3 accuracy, the SVM, LSTM, and NMT models achieved 95.64%, 95.72%, and 95.60% for experiment 1, 95.75%, 95.67%, and 95.69% for experiment 2, and 95.88%, 95.93%, and 95.06% for experiment 3, respectively.

**Conclusions:**

This study demonstrates the feasibility of creating an automated anesthesiology CPT classification system based on NMT techniques using surgical procedure text and preoperative diagnosis. Our results show that the performance of the NMT-based CPT prediction system is equivalent to that of the SVM and LSTM prediction models. Importantly, we found that including preoperative diagnoses improved the accuracy of using the procedure text alone.

## Introduction

### Background

In 2017, the administrative costs between insurers and providers in the United States were excessively high, totaling US $812 billion, US $2497 per capita, and representing 34.2% of the total health expenditures [1]. Billing and insurance-related expenditures in the United States are twice that of Canada and the Netherlands and quadruple that of Sweden [2-4]. Studies by Himmelstein et al [1,2] identify administrative costs as one of the major drivers of high health care expenditures in the United States and support the necessity of cost reduction. Furthermore, billing and insurance-related activities, a subset of administrative costs, are between 8.4% and 13.9% of the total revenue [5]. There is an opportunity to reduce these administrative costs associated with billing assignments using automation, leveraging machine learning and natural language processing techniques.

To analyze the administrative costs, Tseng et al [6] and O’Malley et al [7] illustrated the process of billing activities (known as *the life of a bill*) from the initial appointment to the time when payment was received. In this process, physicians are first involved in billing activities related to clinical services, even before the patient visit. Next, professional and hospital billing activities occur after the patient visit, where professional coders and billing management teams are involved in coding and managing claims that involve extensive and laborious hospital chart reviews. In particular, Tseng et al [6] found that billing and insurance-related activities carried out by physicians were between US $6.36 per primary care visit and US $51.20 per inpatient surgical procedure, which is 11%-31% of the total administrative costs. Similarly, professional and hospital billing teams’ administrative costs range from US $4.22 to US $45.55 per procedure, accounting for 3%-36% of the total administrative costs.

Automating the coding process could also improve otherwise high rates of medical coding errors. According to the Comprehensive Error Rate Testing report published in the Centers for Medicare and Medicaid Services, Medicare’s improper payments were US $36.2 billion in 2017, 9.5% of the total Medicare payment. The common reasons for improper payments were insufficient documentation errors (64.1%), medical necessity errors (17.5%), incorrect coding errors (13.1%), and no documentation errors (1.7%) [8]. With regard to the effort of evaluating coding errors, numerous studies have associated physicians’ limited training and knowledge of billing and insurance-related activities with high errors [9-12]. King et al [10] showed that family physicians’ coding accuracy was 52% for established patients and 17% for new patients, as established patients were undercoded, thus failing to report the full services provided, whereas new patients were overcoded, an abuse in reporting medical services not actually performed.

In our study, we developed a two-step neural machine translation (NMT) model to automate current procedural terminology (CPT) coding. This NMT-based model translates manually entered surgical procedure text by a surgeon into a standard CPT description in step 1 and then calculates similarity scores to match the best CPT code in step 2. A single-step automated billing system estimates the likelihood of multiple classes, whereas standardized CPT descriptions from step 1 of the NMT-based model have potential use, in addition to this CPT prediction task. The standardized text from this NMT-based model can improve communication efficiency between physicians and hospital professionals in medical coding processes and ultimately reduce administrative costs by aiding in CPT code classification and reducing medical coding errors. In this study, we demonstrate both the translation performance and CPT prediction accuracy.

### Related Work

Advanced machine learning methods have been developed to automate the manual classification of medical codes, including the International Classification of Diseases (ICD) [13-21] and CPT [22-24] codings. In these previous efforts, researchers used narrative clinical notes along with structured data elements to develop machine learning classification algorithms. For traditional machine learning algorithms, Koopman et al [14] proposed a binary support vector machine (SVM) classifier for multiple ICD-10 codes using n-gram features. Perotte et al [13] developed a hierarchy-based SVM by leveraging the hierarchical structure of the ICD-9 codes. Denck et al [16] showed an ensemble of classifier chains to predict billing codes using MRI log data. Virginio and dos Reis [21] and Wu et al [25] used the SVM model to discuss imbalanced data in medical billing data.

With advanced deep neural networks and natural language processing techniques, researchers can apply clinical notes without extensive preprocessing of raw narrative clinical text into a recurrent neural network or convolutional neural network. Xu et al [17] evaluated an ensemble model where unstructured, semistructured, and structural data were trained on text-based convolutional neural networks, bidirectional long short-term memory (LSTM), and decision tree for ICD prediction. Shi et al [26] developed a hierarchical LSTM model with attention techniques to classify clinical notes into ICD codes. Wang et al [27] proposed a label embedding attentive model using label-attentive text representation to improve text classification and applied it to predict ICD codes using clinical notes. Rios and Kavuluru [28] improved the accuracy of ICD prediction by transferring learning with PubMed biomedical abstracts.

NMT has emerged as a state-of-the-art machine learning method for translating text between human languages. The Transformer-based [29] NMT model uses an encoder-decoder framework with self-attention mechanisms to learn the weights of a translation model and understand the complex relationships between the source and target languages [30-32]. Although NMT models have been adopted in health care to assist health communication such as speech translation or text translation from one language to another [33], these models remain underutilized.

## Methods

### NMT Model Architecture

We developed an NMT-based automated CPT coding system that first translates surgical procedure texts in electronic health records (EHRs) into preferred terms from the Unified Medical Language System (UMLS) [34] and then normalizes the translated preferred term to predict CPT codes. The intuition of this machine translation approach is from text normalization, the process of transforming noncanonical text into a standard form such as medical concept normalization [35-38] where medical terms are assigned to unique concept codes.

**Figure 1:**
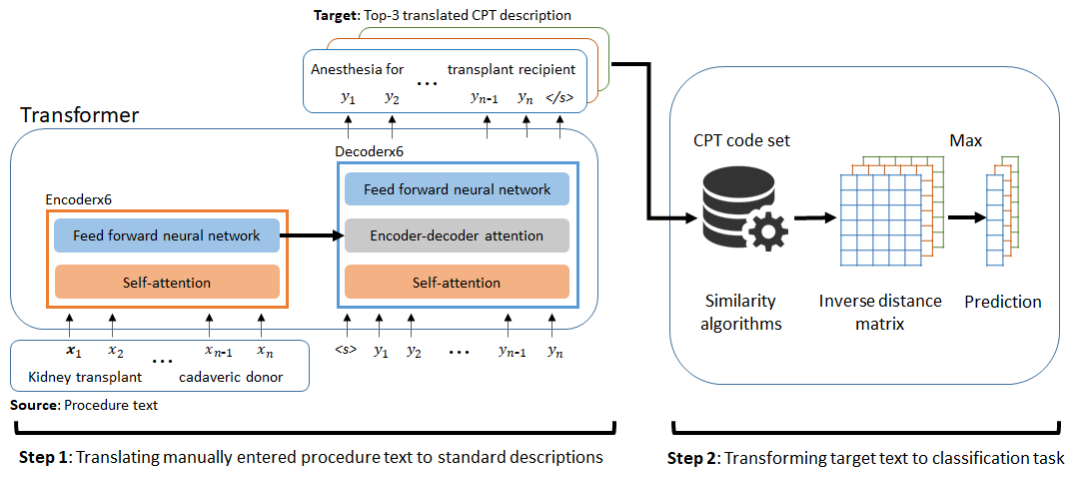
The architecture of an automated current procedural terminology coding system based on the Transformer model. CPT: current procedural terminology.

Within medicine, each surgical procedure contains a surgical procedure text and a preoperative diagnosis entered by a surgeon or surgical resident. After completion of the procedure, surgical and anesthesiology CPT codes were assigned by clinical staff and/or professional medical coders. The manually entered texts are the input source, and the preferred terms of the assigned CPT codes are the output target sentences of the NMT model. In our study, surgical procedure texts and preoperative diagnoses were the inputs of the model to predict CPT codes. The architecture of the NMT-based automated CPT prediction system is shown in Figure 1.

The overall architecture of an NMT-based system is composed of *translation* and *transformation* components. The *translation* component (step 1 in Figure 1) translates surgical procedure text into a standard form using the *Transformer* model [29]. This model uses a set of encoders and decoders using self-attention mechanisms and feed-forwarding neural networks. This Transformer model computes representations between a sequence of source word embeddings, x_i_, and a sequence of target word embeddings, y_i_, trained from the procedure text and the description of the CPT code.

To train the NMT model, the source and target sentences need to be paired between the manually entered procedure text (source) and the preferred terms of the CPT code (target). This is similar to the development of paired bilingual sentences to train a language translation model. Once trained, the NMT model generates multiple candidate translation outputs ranked by a beam search algorithm. The top three target sentences were retained and processed through step 2: *transformation*.

With the three target sentences, the best CPT code was computed in the transformation step using the Levenshtein and Jaccard distances. The distance was computed between a target sentence and the preferred term of all CPT codes in the UMLS. Each sample was compared with the CPT descriptions, and its value was stored in a distance matrix, *X* ∈ 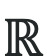
^M x N^, where M is the number of the sample size and N is the number of CPT labels. As closer distances signify better matching to the CPT descriptions, we used an inverse distance matrix, 1/(*X* + 1), to maintain similarity scores for the three target sentences. The final prediction of the CPT code was based on the highest score in the matrix. *X* is an M by N matrix, where M is the number of the sample size and N is the number of the label size.

Distance Matrix = *X* ∈ 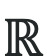
^M x N^**(1)**

Inverse Distance Matrix_i,j_ = 1/(x_i,j_ + 1), i = 1,..., m; j = 1,..., n **(2)**

Prediction_i_ = argmax_j∈_*_J_* (1/(x_i,j_ + 1)), i = 1,..., m; *J* = {1,..., n} **(3)**

The key implication of this two-step NMT-based system is to reframe the translation task to a multiclass classification task using translation and transformation steps. Unlike a single classifier used in other automated coding algorithms [15-17,26,27,39-42], the two-step NMT model translates noncanonical text of human natural language into a normalized form and then transforms the normalized translation into an appropriate CPT code.

### Data

The Multicenter Perioperative Outcomes Group (MPOG) is a nonprofit academic consortium of more than 50 academic and community hospitals across 19 states in the United States and the Netherlands [43,44]. The extent of data in the MPOG is relevant to the field of anesthesiology encompassing perioperative patient care, covering preoperative, intraoperative, and postoperative clinical practice. Through the MPOG infrastructure, a large volume of perioperative data such as patient vital signs, ventilation, medications, laboratory values, and administrative billing data from EHRs at different centers have been systematically aggregated via automated extraction and validated by clinical experts.

**Figure 2:**
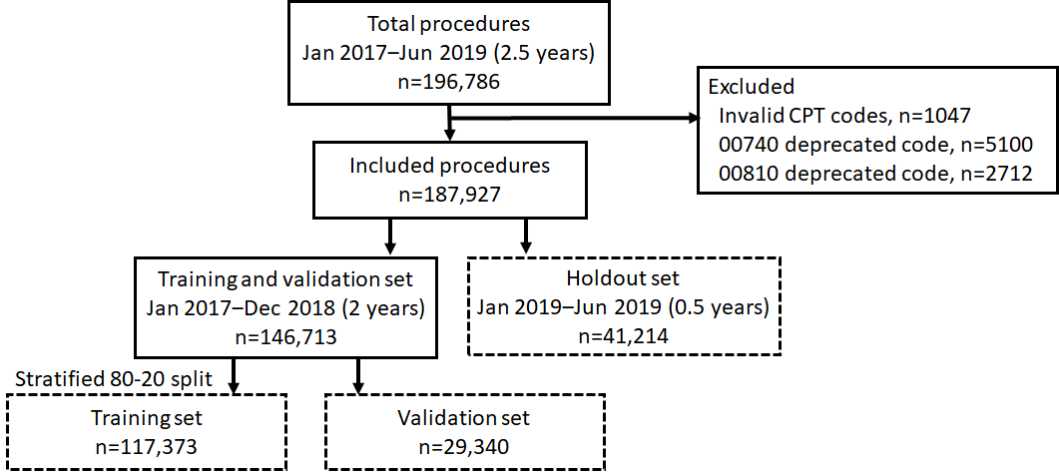
The flowchart of data selection and rules to split the training, validation, and holdout sets. CPT: current procedural terminology.

We collected all operative procedures performed at Michigan Medicine from January 2017 to June 2019 (2.5 years), resulting in 196,786 operative cases. In these data, we found that 10 unique CPT codes were invalid due to typographic errors and two unique CPT codes (00740: anesthesia for upper gastrointestinal procedures; 00810: anesthesia for lower gastrointestinal procedures) were deprecated in 2018 and replaced with newer CPT codes (00731 and 00732 and 00811, 00812, and 00813, respectively). These invalid and deprecated CPT codes (8859 of operative cases), were excluded from the analysis (Multimedia Appendix 1). The total number of operative procedures used to develop and evaluate the machine learning models used in this study was 187,927. Of the 272 anesthesiology CPT codes in the UMLS, we found 269 unique anesthesiology CPT codes in the final data set. A detailed flowchart of the inclusion and exclusion of the data is shown in Figure 2.

### Training, Validation, and Holdout Data Splits

The final number of operative cases used in this study was 187,927. Procedures performed between January 2019 and June 2019 (6 months) were set aside as the holdout set, and procedures performed in 2017 and 2018 (2 years) were used to train and validate the machine learning models.

The implication of splitting the holdout set based on an explicit date before and after 2019 from the training and validation sets is to measure the behavior of predictive models on unseen future data. The uncertainty of the distribution of surgical procedures should remain separate from training to real predictions. This date-based split technique minimizes data leakage and information accidentally shared between the training and holdout sets.

The data used for training and validation were stratified based on CPT codes (labels) and randomly split into 80/20 training and validation sets for model development and hyperparameter tuning. The stratified split between the training and validation sets enables maintenance of the same rate of CPT codes in both data sets, allowing an even split of rare surgical procedures.

The prevalence of the surgical procedures per CPT category in the training, validation, and holdout sets is shown in Table 1. The notable difference between the training and the holdout set is that the percentage of upper and lower abdomen procedures was higher in 2019 (holdout set) than in 2017 and 2018 (training set).

**Table 1:** The prevalence of anesthesiology procedures and services categorized by area of the body in the training, validation, and holdout data sets (N=187,927).

Body part^a^	CPT^b^ codes	Data set^c^, n (%)		
		Training set^d^ (n=117,373)	Validation set^d^ (n=29,340)	Holdout set^e^ (n=41,214)
Head	00100-00222	27,863 (23.74)	6964 (23.73)	9110 (22.10)
Neck	00300-00352	9403 (8.01)	2352 (8.01)	2822 (6.84)
Thorax (chest and shoulder)	00400-00474	5554 (4.73)	1388 (4.73)	1710 (4.14)
Intrathoracic	00500-00580	8781 (7.48)	2194 (7.47)	2903 (7.04)
Spine and spinal cord	00600-00670	2606 (2.22)	651 (2.21)	883 (2.14)
Upper abdomen	00700-00797	12,083 (10.29)	3022 (10.29)	5518 (13.38)
Lower abdomen	00800-00882	13,338 (11.36)	3334 (11.36)	6547 (15.88)
Perineum	00902-00952	9853 (8.39)	2464 (8.39)	3001 (7.28)
Pelvis (except hip)	01112-01173	551 (0.46)	137 (0.46)	194 (0.47)
Upper leg (except knee)	01200-01274	2390 (2.03)	595 (2.02)	674 (1.63)
Knee and popliteal area	01320-01444	2629 (2.24)	658 (2.24)	792 (1.92)
Lower leg (below knee)	01462-01522	2117 (1.80)	530 (1.80)	606 (1.47)
Shoulder and axilla	01610-01680	2084 (1.77)	520 (1.77)	668 (1.62)
Upper arm and elbow	01710-01782	785 (0.66)	196 (0.66)	192 (0.46)
Forearm, wrist, and hand	01810-01860	3035 (2.58)	757 (2.58)	870 (2.11)
Radiological procedure	01916-01936	8709 (7.42)	2179 (7.42)	2829 (6.86)
Burn excisions or debridement	01951-01953	505 (0.43)	126 (0.42)	105 (0.25)
Obstetric	01958-01969	4969 (4.23)	1243 (4.23)	1722 (4.17)
Other procedure	01990-01999	118 (0.10)	30 (0.10)	68 (0.16)

^a^Anesthesiology current procedural terminology codes are categorized based on the area of the body part.

^b^CPT: current procedural terminology.

^c^The percentage may not sum up to 100 because of rounding.

^d^The training and validation sets were stratified and split to maintain the same prevalence of procedures.

^e^The holdout set is new data collected for 6 months to prevent data leakage.

Two types of data, the *operative procedure text* and *preoperative diagnosis,* were extracted from the MPOG. The operative procedure text is a short description of the surgical operation, and the preoperative diagnosis is a disease diagnosis specific to the patient and correlates with the planned surgical procedure. Both text fields are typically entered manually into an EHR system by a surgeon or surgical resident before the surgery. In our training data set, the average length of the procedure text was 5.12 (SD 3.57) words, and the average length of the preoperative diagnosis was 4.12 (SD 2.5) words.

In addition to free-text narratives in EHRs, standard forms of CPT descriptions were extracted from the preferred terms in the UMLS. As the UMLS preserves canonical presentations of medical concepts, CPT codes and preferred terms maintain a one-to-one association. The average length of the preferred terms in the UMLS was 13.23 (SD 6.44) words.

The basic descriptive statistics of the data sets in Table 2 show the number of tokens (words) recorded in the procedure text, preoperative diagnosis, and preferred terms of CPT from the UMLS in each training, validation, and holdout sets. Table 2 also shows the large variation of the manually entered procedure text with 13,847 unique procedure texts in the training set for the 252 unique CPT codes. This indicates the challenge of CPT coding, even with a single data field, to find the most appropriate CPT code.

**Table 2:** Descriptive statistics of operative procedure text, preoperative diagnosis, and preferred terms.

Data set	Number of unique CPT^a^ codes	Number of unique procedure texts	Number of tokens in procedure text^b^				Number of tokens in preoperative diagnosis^b^			Number of tokens in preferred terms^b^	
			Mean (SD)	Range		Mean (SD)		Range	Mean (SD)		Range
Training	252	13,847	5.12 (3.57)	1-60		4.12 (2.5)		0-15	13.23 (6.44)		3-46
Validation	231	6012	5.15 (3.64)	1-51		4.11 (2.5)		0-13	13.23 (4.45)		3-41
Holdout	224	6731	4.98 (3.52)	1-60		4.01 (2.52)		0-13	13.24 (6.18)		3-41

^a^CPT: current procedural terminology.

^b^The unit of descriptive statistics is token (word).

### Models: SVM, LSTM, and NMT

The NMT-based automated CPT prediction system is supported by the encoder-decoder methods proposed in the text normalization study by Lusetti et al [45] and the self-attention encoder and decoder in OpenNMT [46], which uses Google’s base Transformer model and hyperparameters as shown in Multimedia Appendix 2. This NMT-based system generates three translated descriptions of CPT codes and transforms this translation into CPT prediction using similarity algorithms. Although the system contains translation and transformation components that can be evaluated separately, the primary focus of the systems is to measure the performance of predicting CPT codes based on the NMT model to assist physicians and professional coders. Thus, the outcome measurement is based on the accuracy of the CPT prediction and is compared with other classifiers that were conducted in other studies.

We selected the SVM and LSTM models as the baseline models often adopted in medical billing prediction and as developed in our previous study [24]. For SVM model development, we used the standard Scikit-learn packages in Python (Python Software Foundation) and applied grid search cross-validation for training and tuning hyperparameters. The input features of the SVM model were bigrams extracted from the training data and weighted using the term frequency-inverse document frequency. Owing to the large size of features, we limited the inputs to terms that appeared at least four times in the procedure text and at least 15 times in the combined text of procedure and preoperative diagnosis text.

For the LSTM model development, a sequence of words from the procedure text and preoperative diagnosis text in the training data was fed into the embedding layer. The embedding layer then converted each word in the sequence to a vector representation using a Word2Vec model pretrained on PubMed, PubMed Central, and Wikipedia [47]. The LSTM model was trained on this sequence of vector representations and returned a hidden vector from each state that was passed through a fully connected layer. A final softmax layer was then used to predict the final label. The configurations and hyperparameters of the models are shown in Multimedia Appendix 2.

### Experimental Settings

We designed three experimental settings for CPT code prediction with different types of data to evaluate the performance of NMT, SVM, and LSTM machine learning models.

Experiments 1 and 2 used surgical procedure texts as an input to machine learning models with no demographics or clinical information. As misspellings and use of acronyms in manual data entry are common, raw surgical procedure texts without preprocessing were used for experiment 1 and with preprocessing for experiment 2. The purpose of this experiment was to evaluate the process of text normalization from noncanonical procedure text into a single standard description using a translational model. As a CPT code is primarily determined on surgical procedure text, we focused on the association between procedure texts and standard target sentences in the UMLS by restricting the input of the models. The average length of words of the CPT description in the UMLS is 2.6 times longer than the average length of the procedure text, as shown in Table 2. The performance of top-1 (the best prediction) and top-3 (within the top 3) accuracy will be compared between machine learning models.

Experiment 3 was designed to introduce a preoperative diagnosis in the prediction model. Instead of just the preoperative text, the preoperative diagnosis is also appended to the input of the models. The rationale of this experiment is to evaluate the impact of indirect information on CPT code prediction over that in experiment 2. This incorporation is logical, as surgical procedures can be listed with the same procedure text but coded differently because of the diversity in patient diagnosis. Experiment 3 aimed to determine if this multicoded issue could be mitigated by including the preoperative diagnosis in the models. Especially for the NMT model, where the source and target sentences are paired with the same information, the preoperative diagnosis included in the source would be extraneous. The average length of the combination used in the models was 9.1 (SD 4.55) words.

### Preprocessing

Before training the models, the operative procedure and the preoperative diagnosis text were subjected to a series of preprocessing steps, including removing stop words, trimming white spaces, lowering cases, lemmatizing, correcting misspelled medical words, and expanding acronyms (Multimedia Appendix 3). The spelling correction and acronym expansion were manually reviewed, maintained, and applied to curated preoperative texts and the preoperative diagnosis. The curated version of the procedure text in combination with the preoperative diagnosis was used as input for model development for experiments 2 and 3.

### Evaluation and Performance Metrics

The primary performance metric used to evaluate our system is the accuracy of the documented CPT codes within the MPOG database. We defined two accuracies: top-1 is the accuracy where the true CPT code matches with the first top (most probable) model predicted CPT code, whereas top-3 is the accuracy where the true CPT code matches with any one of the top three most probable CPT codes predicted by the models. The top-1 and top-3 accuracies have different implications. The top-1 accuracy presents the single best-predicted CPT code to measure how models would accurately perform on the medical billing code assignment without additional human effort. In contrast, the top-3 accuracy provides the top three most probable CPT codes, reducing selection by the administrative staff to three probable choices. With this rationale, both top-1 and top-3 translated standard forms can be used to assist and audit billing codes by suggesting appropriate options in real time and improving the efficiency of communication between health care professionals.

The top-1 and top-3 accuracies were evaluated for both validation and holdout sets for the three machine learning models. During evaluation, we repeated the experiment 500 times, bootstrapping 20,000 samples with replacements and selecting the best model after tuning the hyperparameters on the validation set. The SVM and LSTM models returned the final output with the predicted probabilities of the 272 CPT labels. The three highest probabilities were selected for top-3 accuracy. As the output of the NMT model is translation sentences, the top three translations were used for the top-3 accuracy evaluation.

### Evaluation of Imbalanced Labels

Class imbalance in medical coding is inevitable because of the nature of hospital services [21,25]. Regular hospital services will show more often in EHRs than in rare cases. For algorithm development, the lack of data in minority classes often creates potential issues and limitations. For example, minority classes that are often underevaluated are critical for patient care. A general approach is to exclude the minority from the data set [17,26,27] and analyze the results on majority classes. It may be valid for evaluating algorithm performance but may raise concerns about implementing the algorithm in clinical settings.

**Figure 3:**
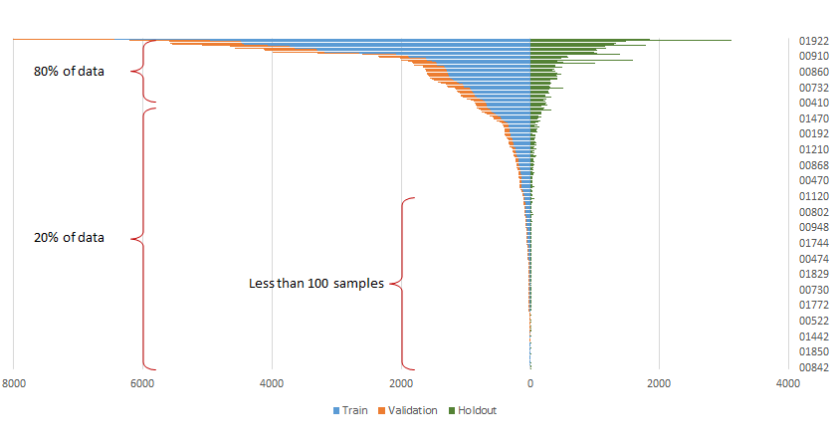
The distribution of current procedural terminology codes in the training, testing, and holdout sets, sorted by most to least frequent codes.

Figure 3 shows the CPT label distribution in our data set between the training, validation, and holdout sets. The top 52 CPT codes accounted for 80.2% (94,216/117,373) of surgical procedures at the University of Michigan, and 220 CPT codes shared the remaining 19.7% (23,157/117,373) of cases. Of these 220 CPT codes, 132 CPT codes, that is, 48.5% (132/272) of the total anesthesiology CPT codes, had less than 100 training samples for 2 years of hospital services.

For the sensitivity analysis of imbalanced data, the anesthesiology CPT codes were split into 10 groups with a mean of 25 (SD 1.3) codes per group to represent the different sizes of samples in the training set (Multimedia Appendix 4). We evaluated and compared the performance of each group between the NMT, SVM, and LSTM models.

## Results

### Top-1 and Top-3 Accuracy

The top-1 and top-3 accuracies of the NMT, SVM, and LSTM models over three experiments are summarized in Table 3. For top-1 accuracy, the NMT model yielded the highest top-1 accuracy for both experiments 1 and 2 at 81.64% and 81.71% when compared with the SVM model (81.19% and 81.27%) and the LSTM model (80.96% and 81.07%), respectively. The SVM model yielded the highest top-1 accuracy of 84.30% in experiment 3, followed by the LSTM (83.70%) and NMT (82.80%) models.

**Table 3:** Performance comparison of support vector machine, long short-term memory, and neural machine translation models with raw, curated procedure text and combined, curated procedure text and preoperative diagnosis.

Model		Top-1 accuracy^a^ (95% CI)		Top-3 accuracy^b^ (95% CI)	
		Validation set	Holdout set	Validation set	Holdout set
**Experiment 1: raw procedure text^c^**					
	SVM^d^	83.61 (83.07-84.16)	81.19 (80.63-81.75)	97.56 (97.36-97.76)	95.64 (95.35-95.93)
	LSTM^e^	81.86 (81.33-82.40)	80.94 (80.42-81.46)	95.38 (95.06-95.71)	95.72 (95.44-95.99)
	NMT^f^	81.68 (81.14-82.21)	81.64 (81.11-82.18)	95.27 (94.96-95.58)	95.60 (95.30-95.89)
**Experiment 2: curated procedure text^g^**					
	SVM	83.38 (82.85-83.90)	81.27 (80.72-81.82)	97.45 (97.23-97.67)	95.75 (95.47-96.04)
	LSTM	81.81 (81.28-82.34)	81.07 (80.54-81.59)	95.32 (95.00-95.64)	95.67 (95.40-95.95)
	NMT	81.72 (81.18-82.26)	81.71 (81.18-82.24)	95.41 (95.11-95.71)	95.69 (95.40-95.98)
**Experiment 3: curated procedure text and preoperative diagnosis^h^**					
	SVM	87.62 (87.15-88.09)	84.30 (83.81-84.79)	99.16 (99.03-99.29)	95.88 (95.60-96.15)
	LSTM	83.52 (83.00-84.04)	83.70 (83.20-84.20)	95.82 (95.53-96.12)	95.93 (95.65-96.20)
	NMT	82.43 (81.90-82.96)	82.80 (82.31-83.29)	94.75 (94.44-95.06)	95.06 (94.77-95.35)

^a^Top-1 accuracy is the accuracy of models on the best current procedural terminology code predicted, equivalent to the F1-score micro.

^b^Top-3 accuracy is the accuracy of models if the true current procedural terminology is within the top three best codes predicted.

^c^Raw procedure text is manually entered by physicians without text preprocessing.

^d^SVM: support vector machine.

^e^LSTM: long short-term memory.

^f^NMT: neural machine translation.

^g^Curated procedure text is cleaned text with preprocessing techniques.

^h^The curated procedure text is concatenated with preoperative diagnosis to training models for current procedural terminology prediction.

For top-3 accuracy, the SVM, LSTM, and NMT models achieved 95.64%, 95.72%, and 95.60% accuracy for experiment 1 and 95.75%, 95.67%, and 95.69% accuracy for experiment 2, respectively. As expected, all three models performed significantly higher when comparing top-3 accuracy with top-1 accuracy. The improvement rate of top-3 accuracy over top-1 accuracy in experiment 2 was 17.8%, 18.23%, and 17.1% for the SVM, LSTM, and NMT models, respectively.

By combining the preoperative diagnosis data in experiment 3, the three models improved top-1 accuracy when compared with experiments 1 and 2. The SVM model obtained the most significant enhancement by achieving 84.30% top-1 accuracy, a 3.7% increase from experience 2. The top-1 accuracy of the LSTM and NMT model was 83.70% and 82.80%, which increased by 3.4% and 1.3%, respectively. For top-3 accuracy, the SVM and LSTM model achieved 95.88% and 95.93%, which is 0.1% and 0.3% enhancement from experiment 2, respectively. The top-3 accuracy of the NMT model was 95.06%, which was reduced by 0.6%.

### Accuracy by Training Sample Size

We further examined the results in Table 3 based on the training sample size to understand the effect of imbalanced labels. A total of 272 CPT codes were split into 10 groups, and each group contained approximately 27 CPT codes based on the training sample size. For example, group 1 contained CPT codes ≤2 samples in the training set, and group 2 included CPT codes that have ≥2 samples and ≤6 samples. As the group number increased, the size of the samples increased.

**Figure 4:**
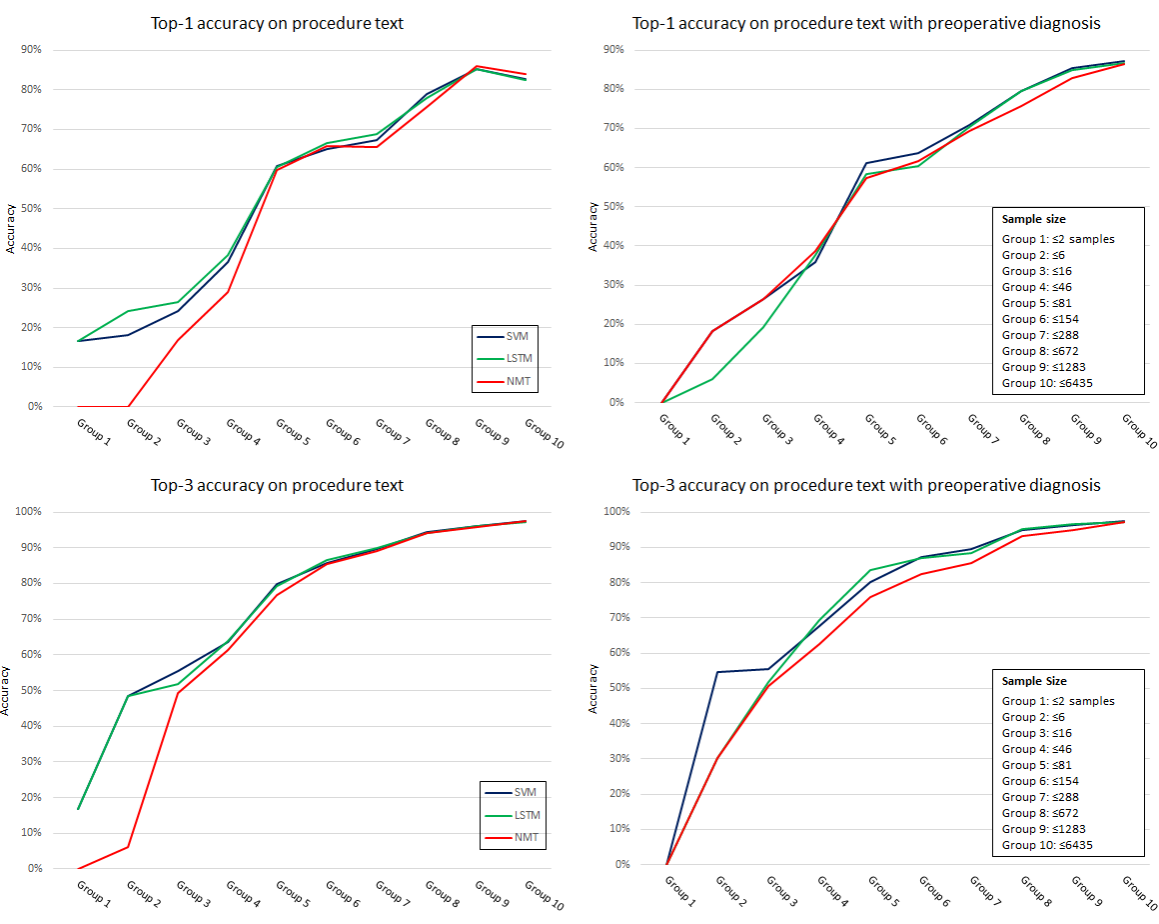
The top-1 and top-3 accuracy comparison based on the training sample size. LSTM: long short-term memory; NMT: neural machine translation; SVM: support vector machine.

The effect of imbalanced labels on performance is illustrated in Figure 4. The top two-line charts show the top-1 accuracy on curated procedure text (top-left) and combined, curated procedure text with a preoperative diagnosis (top-right). The bottom two-line charts show the top-3 accuracy on curated procedure text (bottom-left) and combined text (bottom-right). The top-1 and top-3 accuracies of the NMT model using procedure text were better in group 10 (training sample size >1283) than in other models, but the performance slowly decreased as the sample size decreased. Details on the performance of each imbalanced label are provided in Multimedia Appendix 4.

### NMT Result

**Figure 5:**
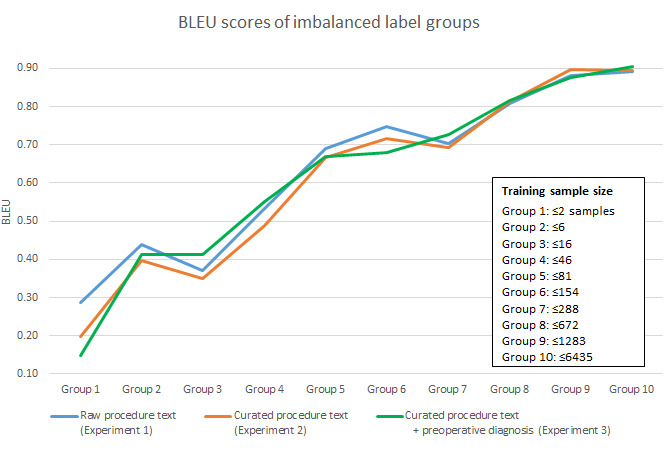
Bilingual Evaluation Understudy scores of imbalanced labels for translating manually entered procedure text into preferred terms in step 1 of the neural machine translation–based model. BLEU: Bilingual Evaluation Understudy.

In addition to the CPT prediction accuracy, the translation performance of imbalanced labels from groups 1 to 10 is summarized in Figure 5. As the sample size increased, the Bilingual Evaluation Understudy (BLEU) scores from all three experiments were significantly improved, and group 10 was close to 0.9. This means that the translation of manually entered procedure texts was close to the preferred terms in the UMLS. The overall BLEU scores of experiments 1, 2, and 3 on the holdout set were 0.872, 0.895, and 0.904, respectively. The detailed BLEU scores are provided in Multimedia Appendix 5.

Examples of translated sentences from our NMT model are presented in Table 4. This table includes the input source text, the output target text, and the gold standard translation used for the NMT model to translate manually entered procedure texts and preoperative diagnoses into standard CPT descriptions. We distinguished the preoperative diagnosis from the procedure text by underlining the source text. The example of 01220 (group 5) demonstrates how additional preoperative diagnosis information ratified the machine translation from manually entered procedure text.

**Table 4:** The neural machine translation–based model’s source (input) and target (output) translation examples from the holdout data set. The translation task is to convert manually entered procedure text in electronic health records to preferred terms in the Unified Medical Language System.

Current procedural terminology code		Current procedural terminology description^a^	Example source text^b^	Target text^c^
**Experiment 1 and 2 with procedure text**				
	00868 (group 7)	Anesthesia for extraperitoneal procedures in lower abdomen, including urinary tract; renal transplant (recipient)	Right kidney transplant cadaveric donor	Anesthesia for extraperitoneal procedures in lower abdomen including urinary tract renal transplant recipient
	01610 (group 9)	Anesthesia for all procedures on nerves, muscles, tendons, fascia, and bursae of shoulder and axilla	Midline wide local excision melanoma iolm sln axillary inguinal	Anesthesia for all procedures on nerves muscles tendons fascia and bursae of shoulder and axilla
	00142 (group 10)	Anesthesia for procedures on eye; lens surgery	Left phacoemulsification intraocular lens	Anesthesia for procedures on eye lens surgery
	00104 (group 10)	Anesthesia for electroconvulsive therapy	Electroconvulsive therapy	Anesthesia for electroconvulsive therapy
	01220 (group 5)	Anesthesia for all closed procedures involving upper two thirds of femur	Left closed reduction cast application	Anesthesia for all closed procedures involving hip joint
**Experiment 3 with a combined procedure text and preoperative diagnosis (underlined)^d^**				
	01402 (group 8)	Anesthesia for open or surgical arthroscopic procedures on knee joint; total knee arthroplasty	Right total knee arthroplasty knee arthritis	Anesthesia for open or surgical arthroscopic procedures on knee joint total knee arthroplasty
	01922 (group 10)	Anesthesia for noninvasive imaging or radiation therapy	Magnetic resonance imaging cardiac anesthesia hypoplastic left heart syndrome	Anesthesia for noninvasive imaging or radiation therapy
	00402 (group 9)	Anesthesia for procedures on the integumentary system on the extremities, anterior trunk, and perineum; reconstructive procedures on breast (eg, reduction or augmentation mammoplasty, muscle flaps)	Left breast tissue expander placement unilateral tissue expander infection	Anesthesia for procedures on the integumentary system on the extremities anterior trunk and perineum reconstructive procedures on breast eg reduction or augmentation mammoplasty muscle flaps
	00812 (group 10)	Anesthesia for lower intestinal endoscopic procedures, endoscope introduced distal to duodenum; screening colonoscopy	Colonoscopy anesthesia encounter screening malignant neoplasm colon	Anesthesia for lower intestinal endoscopic procedures endoscope introduced distal to duodenum screening colonoscopy
	01200 (group 5)	Anesthesia for all closed procedures involving hip joint	Left closed reduction cast application left prosthetic hip dislocation	Anesthesia for all closed procedures involving hip joint

^a^The current procedural terminology description is the *gold standard* translation.

^b^Source text is an example of the preprocessed input of the neural machine translation model.

^c^The target text is the translated sentence from the neural machine translation model.

^d^The preoperative diagnosis in the combined text is underlined to distinguish it from the procedure text.

## Discussion

### Principal Findings

In this study, we demonstrated the feasibility of predicting anesthesiology CPT codes using a two-step machine translation approach. Our results indicate that the top-1 and top-3 accuracies of the NMT-based model were equivalent to those of the SVM and LSTM models using procedure texts. We also demonstrated that the use of additional information, such as preoperative diagnosis, improves SVM, LSTM, and NMT model performance. Finally, we illustrated that imbalanced labels caused by low relative sample sizes negatively affected model accuracy.

We demonstrated the utility of machine learning models for use in medical billing applications. The automated CPT prediction systems developed in this study can improve the accuracy of medical billing coding by decreasing manual data reviews and coding errors. Although 81% accuracy for the top-1 assignment remains low, the top-3 accuracies are above 95%. Therefore, recommendations from top-3 accuracy could be used by physicians and professional billing teams to improve coder accuracy and potentially reduce medical coding assignment and processing times. Translational models have the ability to increase communication efficiency between physicians and billing teams by normalizing the inputs used for assignments. Creating CPT prediction systems can also allow increased auditing efforts to find simple errors in cases that were initially undercoded or overcoded.

The addition of preoperative diagnoses to procedure texts in experiment 3 increased the top-1 accuracy of the SVM, LSTM, and NMT models by 3.7%, 3.2%, and 1.3%, respectively, when compared with experiment 2. The increased accuracy across all three models illustrates the importance of including preoperative diagnoses. For example, several cases may contain similar generic procedure text, such as “exploratory laparotomy,” but entail different operative characteristics requiring differences in billing assignment. By including diagnoses, the models could identify subtle differences beyond the procedure text alone. In addition, there may be significance in the higher improved accuracies for the SVM and LSTM models when compared with the NMT model. The SVM and LSTM models use concatenated procedure text and preoperative diagnosis to predict a CPT code, whereas the NMT-based model uses a paired source and target sentence where the source is concatenated with procedure text and preoperative diagnosis and translated into the target, the preferred term of the CPT code.

This study showed a strong association between the performance of the models and the size of the training samples (Figure 4). When the sample size is above 81 (group 6 or above), all three models’ performance is above 60% for top-1 accuracy and above 75% for top-3 accuracy, which is a significant increase compared with smaller sample sizes. Although all three models performed similarly, the NMT model was more sensitive to the sample size. Comparatively, the NMT model showed a strong performance in the larger sample size (groups 9 and 10) but lower performance when the sample size was smaller than 81 (group 5 or below). This may be due to the complex NMT methods based on the Transformer model compared with the SVM and LSTM models.

The NMT model can be applied in medicine beyond billing. Any free-text entries that often include human or systematic errors can be translated into a standard description as a normalization task, mapping clinical terms in medical notes to a standardized vocabulary. Under the recent effort of clinical entity normalization [36,37], our approach using NMT can be utilized for normalization tasks. This approach offers many advantages: (1) the translated sentence is transparent for clinicians and researchers to identify whether NMT works, allowing the model to be adjusted based on their feedback. (2) Human feature engineering effort is minimal as long as the source and target sentences are paired in model training.

This study has demonstrated the use of creating an NMT-based automatic anesthesiology CPT classification system. Its performance is equivalent to that of the SVM and LSTM models and presents itself as another method for machine learning applications in medicine.

### Study Limitations

This study has several important limitations that must be considered:

1. The models in this study were developed and evaluated using anesthesiology CPT codes from data collected for about 2.5 years. The holdout data comprised only 6 months, from January to June 2019. Although the proportion of CPT codes in the holdout set is similar to the training data, there is still a risk of hidden seasonal effects or trends in rare cases that may not stand out on initial interpretation.
2. Although limiting to two features (operative procedure text and preoperative diagnosis), it allows us to evaluate the translation performance from manually entered text to standard form. This may not achieve the best CPT prediction performance. Some complex examples, such as multiple CPT codes assigned to the same procedure text, may not convey enough information with two features. In addition, lower-frequency codes, as shown in Figure 4, may require careful assessment for CPT prediction.
3. The Centers for Medicare and Medicaid Services reported about Medicare’s improper payments in the Comprehensive Error Rate Testing report. This implies that our data set may contain inherent medical coding errors [10,48]. To reduce these coding errors, a manual review of CPT codes by coding experts is required to enhance data quality before training models.
4. There are residual CPT codes for procedures when no equivalent or limited documentation is available. The description of the residual codes contains “...not otherwise specified.” These residual codes often lead to undercoding, which fails to capture all clinical procedures and reinforce models to learn undercoding behaviors and negatively affects prediction performance.
5. Anesthesiology CPT codes were chosen for classification because of the limited complexity in assignment. There is one anesthesiology code assigned per operative case, allowing for a multiclass single classification, whereas multiple surgical CPTs are often assigned per operative case. In addition to classification complexity, there is a limited number of CPT codes used for anesthesiology classification compared with surgical CPT codes (<300 vs >5000). The extension of the scope beyond anesthesiology CPT codes requires further evaluation to reconfigure the NMT-based model and to adjust better similarity algorithms.
6. The NMT-based model performed on par with one-step models in terms of accuracy, demonstrating the use of translation models to perform CPT classification. However, without a significant increase in accuracy, the additional processing time may prove significant when applied to larger, more complicated billing classifications, such as ICD and surgical CPT predictions. This may limit the real-world application of translation models for these tasks.
7. The performance of complex models, such as NMT, is more subjective to the small sample size than traditional machine learning models. In Figure 4 and Multimedia Appendix 4, the NMT-based model’s performance is lower with fewer training samples and better when more training samples are available.

### Conclusions

In this study, we demonstrated an automated anesthesiology CPT classification system based on machine translation techniques using surgical procedure text and preoperative diagnosis. The overall results show that the NMT-based CPT prediction model is equivalent to the SVM and LSTM models. Although the NMT-based model was not significantly outperformed, this new approach enables researchers to normalize manually entered clinical text into a standard form for use in classification tasks.

## Appendix

Multimedia Appendix 1 Detail counts of data inclusion and exclusion criteria.

Multimedia Appendix 2 Best hyperparameters for the support vector machine, long short-term memory, and neural machine translation models.

Multimedia Appendix 3 Preprocessing step of the cleaning procedure and preoperative diagnosis text.

Multimedia Appendix 4 Performance analysis of imbalanced labels broken down into 10 groups based on sample size. Experiment 1 is exempt from this analysis because the result is similar to that of experiment 2.

Multimedia Appendix 5 The detailed corpus-level Bilingual Evaluation Understudy scores of imbalanced labels for translating manually entered procedure text into preferred terms on the validation and holdout sets.

## Abbreviations

BCBSM: Blue Cross Blue Shield of Michigan
BLEU: Bilingual Evaluation Understudy
CPT: current procedural terminology
EHR: electronic health record
ICD: International Classification of Diseases
LSTM: long short-term memory
MPOG: Multicenter Perioperative Outcomes Group
NMT: neural machine translation
SVM: support vector machine
UMLS: Unified Medical Language System
