# Supplementary material for: Neural Machine Translation–Based Automated Current Procedural Terminology Classification System Using Procedure Text: Development and Validation Study
Source: JMIR Form Res. 2021 May 26;5(5):e22461. doi: 10.2196/22461 (PMC8190648; doi:10.2196/22461)
Supplement: Multimedia Appendix 1 [file formative_v5i5e22461_app1.docx]

**Multimedia Appendix 1.** Detail Counts of Data Inclusion and Exclusion Criteria

| **Description** | **Inclusion** | **Exclusion** | **Rational** |
| --- | --- | --- | --- |
| Starting Population | 196,786 |  | **2.5yr** (2017-01-01 ~ 2019-06-30) |
| Invalid code: 01967.1 |  | 989 | This is similar as 01967 |
| Invalid code: 01967.A1 |  | 34 |  |
| Invalid code: 01967.A2 |  | 2 |  |
| Invalid code: 0042TNL |  | 5 | Invalid |
| Invalid code: 00160.C1 |  | 2 |  |
| Invalid code: 00300.C1 |  | 1 |  |
| Invalid code: 00400.C1 |  | 1 |  |
| Invalid code: 00402.C1 |  | 2 |  |
| Invalid code: 00802.C1 |  | 1 | Invalid |
| Invalid code: 0502F |  | 5 | Not anesthesia CPT |
| Invalid code: 0466T |  | 2 | Not anesthesia CPT |
| Invalid code: 0449T |  | 3 | Not anesthesia CPT |
| Deprecated code: 00740 |  | 5100 | No need to predict deprecated code, and predict to the new CPT codes;  00740 -> 00731, 00732 |
| Deprecated code: 00810 |  | 2712 | No need to prediction deprecated code, and predict to the new CPT codes;  00810 -> 00811, 00812, 00813 |
| **Total** | 196,786 | 8,859 | **187,927** |
