# Supplementary material for: Neural Machine Translation–Based Automated Current Procedural Terminology Classification System Using Procedure Text: Development and Validation Study
Source: JMIR Form Res. 2021 May 26;5(5):e22461. doi: 10.2196/22461 (PMC8190648; doi:10.2196/22461)
Supplement: Multimedia Appendix 2 [file formative_v5i5e22461_app2.docx]

**Multimedia Appendix 2.** Best Hyper Parameters for SVM, LSTM, and NMT models

| **Model** | **Hyper Parameters** |
| --- | --- |
| **Raw Procedure Text** | |
| SVM | C: 0.5  Dual: True  Loss: squared_hinge  Penalty: L2  TF-IDF: minimum number of words: 4 for preoperative text, and 15 for combined text |
| LSTM | L1 lstm units:100  L2 num units:100  L2 dropout:0.2  Activation: Relu |
| NMT | Embedding_size: 512  Encoder - num_layers: 6  Encoder - num_units: 512  Encoder - num_heads: 8  Encoder - ffn_inner_dim: 2048  Encoder - dropout: 0.4  Encoder - attention_dropout: 0.4  Encoder - relu_dropout: 0.4  Decoder - num_layers: 6  Decoder - num_units: 512  Decoder - num_heads: 8  Decoder - ffn_inner_dim: 2048  Decoder - dropout: 0.4  Decoder - attention_dropout: 0.4  Decoder - relu_dropout: 0.4  Maximum length of input: 150  Gradients accumulation: 8  Beam size: 4 |
| **Curated Procedure Text** | |
| SVM | C: 0.3  Dual: True  Loss function: squared_hinge  Penalty: L2  TF-IDF: minimum number of words: 4 for preoperative text, and 15 for combined text |
| LSTM | L1 lstm units:100  L2 num units:100  L2 dropout:0.2  Activation: Relu |
| NMT | Embedding_size: 256  Encoder - num_layers: 6  Encoder - num_units: 720  Encoder - num_heads: 12  Encoder - ffn_inner_dim: 2048  Encoder - dropout: 0.4  Encoder - attention_dropout: 0.3  Encoder - relu_dropout: 0.4  Decoder - num_layers: 6  Decoder - num_units: 720  Decoder - num_heads: 12  Decoder - ffn_inner_dim: 2048  Decoder - dropout: 0.4  Decoder - attention_dropout: 0.3  Decoder - relu_dropout: 0.4  Maximum length of input: 150  Gradients accumulation: 8  Beam size: 4 |
| **Preop Diagnosis + Curated Procedure Text** | |
| SVM | C: 0.5  Dual: True  Loss: squared_hinge  Penalty: L2  TF-IDF: minimum number of words: 4 for preoperative text, and 15 for combined text |
| LSTM | L1 lstm units:100  L2 num units:100  L2 dropout:0.2  Activation: Relu |
| NMT | Embedding_size: 512  Encoder - num_layers: 6  Encoder - num_units: 512  Encoder - num_heads: 8  Encoder - ffn_inner_dim: 2048  Encoder - dropout: 0.4  Encoder - attention_dropout: 0.4  Encoder - relu_dropout: 0.4  Decoder - num_layers: 6  Decoder - num_units: 512  Decoder - num_heads: 8  Decoder - ffn_inner_dim: 2048  Decoder - dropout: 0.4  Decoder - attention_dropout: 0.4  Decoder - relu_dropout: 0.4  Maximum length of input: 150  Gradients accumulation: 8  Beam size: 4 |
