# Supplementary material for: Neural Machine Translation–Based Automated Current Procedural Terminology Classification System Using Procedure Text: Development and Validation Study
Source: JMIR Form Res. 2021 May 26;5(5):e22461. doi: 10.2196/22461 (PMC8190648; doi:10.2196/22461)
Supplement: Multimedia Appendix 3 [file formative_v5i5e22461_app3.docx]

**Multimedia Appendix 3:** Preprocessing step of cleaning procedure and preoperative diagnosis text.

| **No.** | **Preprocessing step** | **Rationale and Comments** |
| --- | --- | --- |
| **1** | Lowercase | Procedure text and preoperative diagnosis are a short description. We use non capitalized letters. |
| **2** | Strip white spaces | Strip white spaces (spaces, tabs) from the beginning and the end of the text string |
| **3** | Lemmatization | Normalize morphological variants for words by retaining a base or dictionary form of the word (e.g., visiting, visited --> visit) |
| **4** | Remove stopwords | Remove stopwords (common English function words, e.g. a, the, of) and following additional words - must, always, ok, group, phi |
| **5** | Expand acronyms | Expand frequently used acronyms; manually maintained in a file |
| **6** | Correct misspelled | Correct common misspellings; manually maintained in a file |
