# Supplementary material for: Neural Machine Translation–Based Automated Current Procedural Terminology Classification System Using Procedure Text: Development and Validation Study
Source: JMIR Form Res. 2021 May 26;5(5):e22461. doi: 10.2196/22461 (PMC8190648; doi:10.2196/22461)
Supplement: Multimedia Appendix 4 [file formative_v5i5e22461_app4.docx]

**Multimedia Appendix 4:** Performance Analysis of Imbalanced Labels Broken down into 10 groups based on the sample size. Experiment 1 is exempt from this analysis because the result is similar to Experiment 2.

**NMT - Experiment 2. Curated Procedure Text**

|  | **Sample Size Threshold** | **Total Sample Size** | **# of unique CPTs** | **Acc@1 (%)** | **Acc@3 (%)** |
| --- | --- | --- | --- | --- | --- |
| Group 1 | <= 2 | 37 | 26 | 0.0 | 0.0 |
| Group 2 | >2 & <=6 | 119 | 27 | 0.0 | 6.06 |
| Group 3 | >6 & <=16 | 265 | 22 | 16.87 | 49.39 |
| Group 4 | >16 & <=46 | 711 | 25 | 28.89 | 61.33 |
| Group 5 | >46 & <=81 | 1,616 | 26 | 59.84 | 76.70 |
| Group 6 | >81 & <=153 | 2,918 | 24 | 65.85 | 85.55 |
| Group 7 | >153 & <=288 | 5,335 | 25 | 65.59 | 89.15 |
| Group 8 | >288 & <=672 | 11,479 | 26 | 75.64 | 94.15 |
| Group 9 | >672 & <=1283 | 24,758 | 25 | 86.09 | 95.88 |
| Group 10 | >1283 & <=6435 | 70,135 | 26 | 83.89 | 97.52 |

**NMT - Experiment 3. Curated Procedure Text + Preoperative Diagnosis**

|  | **Sample Size Threshold** | **Total Sample Size** | **# of unique CPTs** | **Acc@1 (%)** | **Acc@3 (%)** |
| --- | --- | --- | --- | --- | --- |
| Group 1 | <= 2 | 37 | 26 | 0.0 | 0.0 |
| Group 2 | >2 & <=6 | 119 | 27 | 18.18 | 30.30 |
| Group 3 | >6 & <=16 | 265 | 22 | 26.51 | 50.60 |
| Group 4 | >16 & <=46 | 711 | 25 | 38.67 | 62.66 |
| Group 5 | >46 & <=81 | 1,616 | 26 | 57.40 | 75.86 |
| Group 6 | >81 & <=153 | 2,918 | 24 | 61.59 | 82.53 |
| Group 7 | >153 & <=288 | 5,335 | 25 | 69.21 | 85.60 |
| Group 8 | >288 & <=672 | 11,479 | 26 | 75.61 | 93.22 |
| Group 9 | >672 & <=1283 | 24,758 | 25 | 82.92 | 95.04 |
| Group 10 | >1283 & <=6435 | 70,135 | 26 | 86.45 | 97.20 |

**SVM - Experiment 2. Curated Procedure Text**

|  | **Sample Size Threshold** | **Total Sample Size** | **# of unique CPTs** | **Acc@1 (%)** | **Acc@3 (%)** |
| --- | --- | --- | --- | --- | --- |
| Group 1 | <= 2 | 37 | 26 | 16.67 | 16.67 |
| Group 2 | >2 & <=6 | 119 | 27 | 18.18 | 48.48 |
| Group 3 | >6 & <=16 | 265 | 22 | 24.10 | 55.42 |
| Group 4 | >16 & <=46 | 711 | 25 | 36.44 | 63.56 |
| Group 5 | >46 & <=81 | 1,616 | 26 | 60.65 | 79.92 |
| Group 6 | >81 & <=153 | 2,918 | 24 | 65.06 | 85.78 |
| Group 7 | >153 & <=288 | 5,335 | 25 | 67.20 | 89.54 |
| Group 8 | >288 & <=672 | 11,479 | 26 | 78.98 | 94.32 |
| Group 9 | >672 & <=1283 | 24,758 | 25 | 85.25 | 96.08 |
| Group 10 | >1283 & <=6435 | 70,135 | 26 | 82.79 | 97.36 |

**SVM - Experiment 3. Curated Procedure Text + Preoperative Diagnosis**

|  | **Sample Size Threshold** | **Total Sample Size** | **# of unique CPTs** | **Acc@1 (%)** | **Acc@3 (%)** |
| --- | --- | --- | --- | --- | --- |
| Group 1 | <= 2 | 37 | 26 | 0.0 | 0.0 |
| Group 2 | >2 & <=6 | 119 | 27 | 18.18 | 54.55 |
| Group 3 | >6 & <=16 | 265 | 22 | 26.51 | 55.42 |
| Group 4 | >16 & <=46 | 711 | 25 | 36.00 | 67.56 |
| Group 5 | >46 & <=81 | 1,616 | 26 | 61.26 | 80.12 |
| Group 6 | >81 & <=153 | 2,918 | 24 | 63.72 | 87.12 |
| Group 7 | >153 & <=288 | 5,335 | 25 | 70.76 | 89.54 |
| Group 8 | >288 & <=672 | 11,479 | 26 | 79.67 | 94.95 |
| Group 9 | >672 & <=1283 | 24,758 | 25 | 85.35 | 96.27 |
| Group 10 | >1283 & <=6435 | 70,135 | 26 | 87.26 | 97.32 |

**LSTM - Experiment 2. Curated Procedure Text**

|  | **Sample Size Threshold** | **Total Sample Size** | **# of unique CPTs** | **Acc@1 (%)** | **Acc@3 (%)** |
| --- | --- | --- | --- | --- | --- |
| Group 1 | <= 2 | 37 | 26 | 16.67 | 16.67 |
| Group 2 | >2 & <=6 | 119 | 27 | 24.24 | 48.48 |
| Group 3 | >6 & <=16 | 265 | 22 | 26.51 | 51.81 |
| Group 4 | >16 & <=46 | 711 | 25 | 38.22 | 64.00 |
| Group 5 | >46 & <=81 | 1,616 | 26 | 60.45 | 79.31 |
| Group 6 | >81 & <=153 | 2,918 | 24 | 66.63 | 86.56 |
| Group 7 | >153 & <=288 | 5,335 | 25 | 68.88 | 89.93 |
| Group 8 | >288 & <=672 | 11,479 | 26 | 77.89 | 94.24 |
| Group 9 | >672 & <=1283 | 24,758 | 25 | 85.20 | 96.17 |
| Group 10 | >1283 & <=6435 | 70,135 | 26 | 82.51 | 97.18 |

**LSTM - Experiment 3. Curated Procedure Text + Preoperative Diagnosis**

|  | **Sample Size Threshold** | **Total Sample Size** | **# of unique CPTs** | **Acc@1 (%)** | **Acc@3 (%)** |
| --- | --- | --- | --- | --- | --- |
| Group 1 | <= 2 | 37 | 26 | 0.0 | 0.0 |
| Group 2 | >2 & <=6 | 119 | 27 | 6.06 | 30.30 |
| Group 3 | >6 & <=16 | 265 | 22 | 19.28 | 51.81 |
| Group 4 | >16 & <=46 | 711 | 25 | 37.78 | 69.33 |
| Group 5 | >46 & <=81 | 1,616 | 26 | 58.42 | 83.57 |
| Group 6 | >81 & <=153 | 2,918 | 24 | 60.47 | 87.01 |
| Group 7 | >153 & <=288 | 5,335 | 25 | 70.24 | 88.44 |
| Group 8 | >288 & <=672 | 11,479 | 26 | 79.48 | 95.17 |
| Group 9 | >672 & <=1283 | 24,758 | 25 | 85.00 | 96.65 |
| Group 10 | >1283 & <=6435 | 70,135 | 26 | 86.70 | 97.28 |
