# Supplementary material for: Neural Machine Translation–Based Automated Current Procedural Terminology Classification System Using Procedure Text: Development and Validation Study
Source: JMIR Form Res. 2021 May 26;5(5):e22461. doi: 10.2196/22461 (PMC8190648; doi:10.2196/22461)
Supplement: Multimedia Appendix 5 [file formative_v5i5e22461_app5.docx]

**Multimedia Appendix 5.** The detailed corpus-level BLEU scores of imbalanced labels for translating hand-written procedure text into preferred terms on the validation and holdout set.

|  | **Experiment 1** | | **Experiment 2** | | **Experiment 3** | |
| --- | --- | --- | --- | --- | --- | --- |
| Sample Size | Validation set | Holdout set | Validation set | Holdout set | Validation Set | Holdout set |
| Group 1 | 0 | 0.2865 | 0 | 0.1989 | 0 | 0.1493 |
| Group 2 | 0.3223 | 0.4387 | 0.3379 | 0.3962 | 0.3714 | 0.4118 |
| Group 3 | 0.3768 | 0.3721 | 0.373 | 0.3508 | 0.4216 | 0.4135 |
| Group 4 | 0.576 | 0.5326 | 0.5609 | 0.4882 | 0.5913 | 0.5515 |
| Group 5 | 0.6744 | 0.691 | 0.6292 | 0.6664 | 0.6287 | 0.6693 |
| Group 6 | 0.7745 | 0.7474 | 0.7327 | 0.7178 | 0.7025 | 0.6811 |
| Group 7 | 0.7114 | 0.7031 | 0.6983 | 0.693 | 0.7224 | 0.7261 |
| Group 8 | 0.8156 | 0.8089 | 0.8179 | 0.8133 | 0.8148 | 0.817 |
| Group 9 | 0.8571 | 0.8824 | 0.8949 | 0.8976 | 0.881 | 0.8761 |
| Group 10 | 0.8876 | 0.892 | 0.8873 | 0.8954 | 0.8942 | 0.9038 |
| Overall | 0.8605 | 0.8705 | 0.8688 | 0.8746 | 0.8687 | 0.8766 |
